# HPB News

**DOI:** 10.1155/1989/43732

**Published:** 1989


					HPB Surgery 1989, Vol. 1, pp. 171-172
Reprints available directly from the publisher
Photocopying permitted by license only
1989 Harwood Academic Publishers GmbH
Printed in Great Britain
HPB NEWS
HPB CHAPTER IN SOUTH AFRICA
The south african members have organized a national chapter. It has elected
officers from all 7 university medical schools. The present committee consists of:
J. Terblanche, Cape Town
J.A. Myburgh, Johannesburg
J. Krige, Cape Town
P.C. Bornman, Cape Town
L.C. van Rensburg, Stellenbosch
C.J. Nel, Bloemfontein
J.A. van Wyk, Medunsa
E.M. Barker, Natal
H.H. Lawson, Johannesburg
V.O.L. Karusseit, Pretoria
Chairman and national delegate
Vice-chairman
Secretary/treasurer
Committee member
Committee member
Committee member
Committee member
Committee member
Committee member
Committee member
IHBPA PREVIOUSLY IBA
The above held its annual meeting in Nice last September. The meeting had 452
participants. The membership is 225 and 53 new members were admitted. The
1988/89 council is: D.C. Carter (UK), President; A.R. Moossa (USA), President-
elect; R. Praderi (Uruguay), Vice president; D.L. Carr-Locke (UK), Secretary-
treasurer; J. Garden (UK), Associate secretary; E. Seifert (FRG), Associate
treasurer; J. Toouli, T. Osuga, H. Bismuth, H.C. Peiper, I. Ihse, L.H. Blumgart,
J. Puig La Calle, F. Nakayama, H. Debas, D. d'Amico, E. Van Sonnenberg, J.
Mouiel, council members.
On alternate years (1989, 1991, 1993 etc.) the IHBPA will meet in association
with the World Congress of Surgery under the aegis of the International Society
of Surgery (ISS) and Collegium Internationale Chirurgiae Digestivae (CICD). In
the corresponding alternate years (1990, 1992 etc.), the association will meet in
close proximity to a World or International Congress of Gastroenterology/
Endoscopy. Thus the coming meetings will be 1989: Toronto; 1990: Hong Kong;
1991: Stockholm; 1992: South America. R. Keith is the local organizer in Toronto.
The association wants to achieve greater standards in world clinical and scientific
medicine as an independent high-quality scientific organization offering a unique
multidisciplinary forum for critical discussion of all forms of research in hepatic,
biliary and pancreatic fields.
The Frank Glenn Fellowship, (5000 Swiss Francs), was given to Dr. Giovanni
Aloj for his work: "Relationships amongst bile stasis, sepsis and
choledocholithiasis: clinical and experimental studies."
The meeting in Nice marked the 10thy anniversary of the association.
171
172 HPB NEWS
The address to the secretary-treasurer is:
Dr. D.L. Carr-Locke
Gastroenterology Unit
Leicester Royal Infirmary
Leicester, UK.
Tel. (0533) 541414 ext. 240
WORLD ASSOCIATION OF HPB SURGERY
On December 31st, 1988 the association had 1772 members. The largest member-
ship is in Italy (262 members), USA (99 members), Great Britain (87 members),
and West Germany (73 members).
The 1988/1990 Council consists of Martin Adson (USA), President; Bernard
Langer (Canada), Vice President; Niels van der Heyde (Netherlands), President
elect; Stig Bengmark (Sweden), General Secretary; Robin Williamson (Great
Britain), Treasurer; Miles Little (Australia); Christoph Broelsch (USA); Arthur
Li (Hong Kong); Henry Pitt (USA); John Terblanche (South Africa); Hans Beger
(FRG); Enrique Moreno Gonzales (Spain); Giuseppe Gozzetti (Italy) and
members at large.
The 1988/1990 Scientific Committee is: Christoph Broelsch (USA), Chairman;
Irving Benjamin (Great Britain); Peter Fabri (USA); Dominique Franco (France);
John Ham (Australia); Ken Hobbs (Great Britain); Bengt Jeppsson (Sweden);
Bernd Kremer (FRG); Dr. Lam (Japan); Huug Obertop (Netherlands); Malcolm
Puntis (Great Britain); Johannes Scheele (FRG); Paul Sugarbaker (USA); Onno
Terpstra (Netherlands); T. Tobe (Japan).
The third HPB World Congress will take place in Kensington Town Hall in
London, 4th-8th June 1990. A large national committee consisting of Robin
Williamson, Ken Hobbs, Irving Benjamin, Malcolm Puntis and several others are
working hard to prepare a good meeting. Detailed news about the meeting will
follow in the next issue.
The address to the secretary is:
Professor Stig Bengmark
Box 5003
S-220 05 Lund 5
Sweden
Tel. int. 46-46-171160, telefax int. 46-46-172335.

				

